# Perspective of Turkish society toward autistic individuals: Personal experiences, knowledge, and interaction comfort

**DOI:** 10.1371/journal.pone.0351244

**Published:** 2026-06-08

**Authors:** Gamze Alak

**Affiliations:** Department of Special Education, Kazım Karabekir Faculty of Education, Ataturk University, Erzurum, Turkey; Hacettepe University: Hacettepe Universitesi, TÜRKIYE

## Abstract

Despite increasing awareness of autism spectrum disorder (ASD) and autistic individuals, factors shaping social interactions involving autistic individuals in the general population remain relatively underexplored. This study examined the interrelationships among personal experience, knowledge, and interaction comfort toward autistic individuals among Turkish adults and explored demographic differences. A total of 507 participants (aged 18+) were recruited using snowball sampling, and data were collected via an online survey. Data were analyzed using descriptive, comparative, and correlational analyses, followed by regression-based mediation and moderated mediation models (PROCESS macro). Participants primarily reported indirect experiences with autistic individuals, and both knowledge levels and interaction comfort were relatively high, with significant differences across several demographic characteristics. Interaction comfort was lower in social than in professional settings and varied according to levels of support needs. Knowledge mediated the relationship between personal experience and interaction comfort. The indirect effect was significant only at higher levels of interaction quality. These findings highlight the role of knowledge and interaction quality in shaping interaction comfort and suggest the importance of interventions that promote meaningful and informed interactions. Implications for future research and practice are discussed.

## Introduction

Autism spectrum disorder (ASD) is a neurodevelopmental condition characterized by differences in social communication and interaction, as well as restricted and repetitive patterns of behavior, interests, or activities that typically emerge in early childhood [1,2]. While some characteristics are shared across individuals, there is considerable variability in how these features are expressed, which is reflected in differing levels of support needs [3]. Despite this variability, recent large-scale surveillance studies have reported an increase in the prevalence of ASD [4,5]. However, the reasons for this increase remain unclear and are often attributed to a combination of environmental, biological, and diagnostic factors [6,7]

The fact that prevalence data on ASD are often based on children may lead to the formation of a perception that ASD is primarily a childhood condition [8], which may limit public understanding of ASD as a lifelong condition. However, ASD is a lifelong condition, and individuals continue to require varying levels of support throughout adulthood [8–10]. During the transition to adulthood, many autistic individuals seek opportunities for housing, employment, leisure activities, and social support within their communities [11,12] and participation in these domains plays an important role in overall well-being [13]. However, many individuals encounter barriers related to employment, daily living, and social interaction [14–16]. These barriers may reduce opportunities for social participation and increase the risk of social exclusion and loneliness [17]. In addition, such experiences may be associated with negative self-perceptions, a reduced sense of belonging, and mental health difficulties [18–20]. Furthermore, these experiences may also affect family members, leading to withdrawal from social environments and a decreased quality of family life [21].

Difficulties in social interaction experienced by autistic adults are often explained in terms of individual-level social, cognitive, and behavioral differences [22]. From this perspective, challenges in social interaction are attributed to differences in social communication, and interventions typically focus on developing “social skills” [23]. In this process, autistic individuals are often expected to adapt to neurotypical norms, sometimes by masking autistic characteristics [24]. Although these approaches may offer certain benefits, they have also been associated with negative long-term outcomes, particularly in relation to mental health and self-perception [25]. Moreover, explaining difficulties in social interaction solely in terms of individual characteristics may overlook the inherently interactive nature of social interaction. Social interaction is a bidirectional process in which both parties actively contribute, and differences in communication styles may lead to mutual misunderstandings [23,26]. Thus, autistic individuals may sometimes be perceived as socially distant; however, they often desire social connection and meaningful relationships [27]. These findings suggest that difficulties in social interaction arise not only from individual characteristics but also from mismatches in communication between social partners [23,28].

Understanding the bidirectional nature of social interaction is essential for addressing societal attitudes and structural barriers related to ASD. In collectivist cultures such as Turkey, which tend to emphasize group harmony and shared values, differences may sometimes be approached primarily at the individual level rather than through broader social attitudes and environmental factors [2,24]. Although such perspectives aim to support the social participation of autistic individuals, they may overlook the role of the social environment in shaping interaction experiences. This perspective is consistent with Allport’s [29] contact theory, which suggests that intergroup contact-particularly under conditions such as equal status, cooperation, shared goals, and institutional support-can reduce discrimination. Within this framework, personal experience can be understood as a form of contact that may enhance knowledge about autism and, in turn, contribute to more positive attitudes and greater interaction comfort. However, research indicates that increased contact does not always lead to improved outcomes, and contact alone may not be sufficient to promote meaningful change [30–33]. These findings suggest that not only the presence of contact but also its quality is critical, as positive interaction conditions may strengthen knowledge and facilitate more positive attitudes and greater interaction comfort [34,35].

In addition to contact processes, stigma has been identified as a key factor influencing social interactions. Thornicroft et al. [36] conceptualize stigma as consisting of three interrelated components: lack of knowledge (ignorance), negative attitudes (prejudice), and discriminatory behavior. Within this framework, knowledge about autism can be understood as addressing the “ignorance” component, whereas interaction comfort may reflect attitudes toward autistic individuals. Misinterpretations of behaviors associated with ASD may influence these components negatively, potentially contributing to avoidance, fear, or discriminatory responses [2,37]. From this perspective, the quality of intergroup contact is also likely to matter. Positive and supportive interaction conditions may facilitate the translation of increased knowledge into reduced prejudice and greater interaction comfort, whereas negative contact experiences may reinforce existing biases despite exposure. In response to these challenges, various international and national awareness initiatives have aimed to improve public understanding and attitudes toward autistic individuals [38,39]. In the Turkish context, national policies and educational initiatives play an important role in shaping public awareness and attitudes toward ASD. For example, the National Autism Action Plan, published on December 3, 2016, aims to ensure equal access to services and promote independent participation in all areas of social life [40]. In addition, university-level courses and awareness activities contribute to increasing knowledge and improving public attitudes toward ASD [41]. Taken together, these theoretical perspectives suggest that personal experience may function as a form of contact that influences interaction comfort both directly and indirectly through knowledge, and that this relationship may vary depending on the quality of interaction. Accordingly, this study examines the mediating role of knowledge in the relationship between personal experience and interaction comfort, as well as the moderating role of interaction quality in this indirect relationship.

In line with these theoretical propositions, studies examining societal perspectives toward autistic individuals across different cultures have focused on knowledge levels, attitudes, and interaction comfort, based on the assumption that limited understanding may lead to negative attitudes and social discomfort [2,3,42–47]. These studies indicate that knowledge, attitudes, and interaction comfort vary depending on cultural and socioeconomic factors; individuals in more individualistic and economically developed societies tend to demonstrate higher levels of knowledge and more positive attitudes, whereas findings from more collectivist and lower socioeconomic contexts have been more variable [2,3,8,42,43,45,46,48,49]. In addition, individuals with prior experience with ASD, those with higher levels of education, and women tend to report higher knowledge levels and more positive attitudes [2,8,34,48–50]. However, empirical studies directly testing the theoretically proposed relationships between knowledge, contact, and attitudes remain limited [2,6,11,21,34,48,51]; although existing findings suggest positive associations among these variables, research examining more complex models remains relatively limited. Notably, a limited number of studies indicate that the effect of awareness on attitudes may be indirectly mediated by knowledge, pointing to both direct and indirect relationships among these variables [2].

### The current study

In summary, previous research has primarily focused on contact (e.g., awareness and personal experience), knowledge, and attitudes (or interaction comfort) toward autistic individuals across different cultures. However, these studies have been conducted across a relatively limited range of cultural contexts and often rely on relatively small or specific samples (e.g., students, caregivers, or professionals). Moreover, relatively few studies have examined these variables together and explored their interrelationships. In addition, few studies have investigated whether interaction comfort varies across different levels of support needs. Building on this literature, the present study examines personal experience, knowledge, and interaction comfort toward autistic individuals in Turkey, as well as the relationships among these variables. It also investigates whether these variables differ according to demographic characteristics. This study addresses three research questions: (1) What are Turkish people’s levels of personal experience, knowledge, and interaction comfort regarding autistic individuals? It was expected that, given increasing awareness efforts and legal initiatives, these levels would be relatively high. (2) Do personal experience, knowledge, and interaction comfort differ according to demographic characteristics? Based on previous research, differences across demographic groups were expected. (3) Which variables predict interaction comfort, and are there indirect relationships among them? Drawing on contact theory and stigma theory, as well as prior research suggesting that contact, knowledge, and attitudes are interrelated, it was expected that personal experience and knowledge would be associated with interaction comfort, that knowledge would mediate this relationship, and that interaction quality would moderate this indirect relationship, including interactions involving individuals with varying levels of support needs.

## Methods

### Ethics statement

This study was approved by the Research Ethics Committee of Atatürk University (approval number: E-56785782-050-02.04-200108076). The study was conducted in accordance with the Declaration of Helsinki. All participants provided informed consent electronically prior to participation and were informed about the purpose of the study. Participation was voluntary and anonymous, and only individuals aged 18 years or older were included.

### Participants

In this study, an online survey was administered to individuals aged 18 and above residing across the seven geographical regions of Turkey (Mediterranean, Aegean, Eastern Anatolia, Southeastern Anatolia, Central Anatolia, Black Sea, and Marmara). Because participants from different regions were targeted, a snowball sampling method was employed [52]. This approach facilitated access to a geographically diverse sample but may also limit representativeness and the generalizability of the findings, as participants are more likely to be recruited through existing social networks, which may increase the likelihood of overrepresenting certain demographic groups. Following this procedure, the researcher initially contacted eligible participants from different regions via telephone. These participants were then asked to identify and recruit additional eligible individuals by sharing the survey link through WhatsApp, e-mail, Instagram, or other social media platforms. A total of 677 individuals accessed the survey. Of these, seven declined to participate. Among the 670 participants who provided consent, 42 did not respond to any survey items, including demographic questions. An additional 38 participants completed only the demographic section, and 83 participants did not respond to the interaction comfort items. Therefore, the final sample consisted of 507 participants who completed all relevant measures. Table 1 presents the demographic characteristics of the sample.

**Table 1:** Demographic characteristics of the participants (n = 507).

Demographic Characteristic	f	%
*Gender*		
Female	334	65.9
Male	173	34.1
*Age*		
18-27	146	28.8
28-38	183	36.1
39-49	130	25.6
50-60	39	7.7
61+	9	1.8
*Highest education level completed*		
No education level completed	1	0.2
Primary education	8	1.6
Secondary education	6	1.2
High school	84	16.6
Vocational High School	21	4.1
University	237	46.7
Postgraduate	93	18.3
Doctoral degree	57	11.2
*Geographical region*		
Mediterranean	38	7.5
Eastern Anatolia	278	54.8
Aegean	26	5.1
Southeastern Anatolia	24	4.7
Central Anatolia	36	7.1
Marmara	60	11.8
Black Sea	45	8.9
*The most common type of residence*		
Urban	460	90.7
Rural	47	9.3
*Relationship to the field of special education*		
Yes	143	28.2
No	364	71.8

As shown in Table 1, the sample consisted primarily of female participants, individuals in early adulthood, and participants with higher levels of education. Participants were recruited from all geographical regions of Turkey, with a substantial proportion from the Eastern Anatolia region, and most resided in urban areas. Most participants did not report a relationship with the field of special education.

### Data collection tool

In this study, an online survey originally developed by Jacoby [53] and Jacoby et al. [11] was adapted with permission from the authors. The adaptation process involved minor modifications, including item addition, removal, and revision, as well as the inclusion of explanatory information and adjustments to response options, in order to improve clarity and ensure cultural relevance to the Turkish context. The survey consisted of four sections. The first section included questions on participants’ demographic characteristics. In addition to the variables used in the original questionnaire (gender, age, highest level of education, and type of residence), variables related to geographical region and relationship with the field of special education were added to better capture the contextual characteristics of the sample. Variables such as race/ethnicity and average annual household income were not included due to cultural and contextual considerations. In the Turkish context, social categorizations are less commonly discussed in terms of racial or ethnic distinctions, and income may be considered a sensitive topic, which may influence the accuracy of self-reported data. Furthermore, age was categorized into 10-year intervals, educational levels were detailed into specific categories (e.g., high school, vocational high school, university, postgraduate, and doctoral levels), and residential categories were simplified by excluding less contextually relevant options (e.g., suburban), in order to improve clarity, enhance interpretability, and ensure consistency with the characteristics of the Turkish context. In the second, third, and fourth sections of the survey, participants were asked questions to assess their personal experiences with autistic individuals, their knowledge about autism, and their comfort when interacting with autistic individuals. Each section of the questionnaire is described in detail below.

#### Personal experience.

The personal experience section of the questionnaire consisted of five items, including one check-all-that-apply item and four multiple-choice questions. As shown in Table 2, participants were first asked whether they had heard of autism before; to enhance clarity, the item included an explanatory note indicating that autism is also referred to as autism spectrum disorder, and includes Asperger’s syndrome. Responses were recorded as “yes,” “no,” or “not sure.” Participants who responded “no” received a score of 0 and automatically skipped the remaining items, whereas those who responded “yes” or “not sure” completed four additional questions assessing their personal experience with autistic individuals. These items were designed to capture both the type (Item 2) and amount (Items 3–5) of personal experience, following Jacoby et al. [11].

**Table 2:** Descriptive statistical results regarding personal experience.

Items	Answer Options	f	%
1. Have you heard of autism before? It is also known as autism spectrum disorder and includes Asperger’s syndrome. (n = 507)	Yes	426	84.0
	No	73	14.4
	I am not sure	8	1.6
2. Where did you hear about autism spectrum disorder (please mark all options that apply to you) (n = 434)	I have been diagnosed with autism	1	0.2
	I have a close friend or family member diagnosed with autism	38	8.8
	I have an acquaintance diagnosed with autism	92	21.2
	I saw autism portrayed in the media	183	42.2
	I read books about autism	56	12.9
	I received information about autism at school or at work	148	34.1
	I heard about autism through word-of-mouth	97	22.4
	I had a job working with people diagnosed with autism	42	9.7
3. How many different individuals with a diagnosis of ASD did you meet? These individuals may include acquaintances or characters from a television show. (n = 434)	1	62	14.3
	2	86	19.8
	3-5	124	28.6
	6-9	25	5.8
	10+	51	11.8
	I did not meet anyone with a diagnosis of autism	86	19.8
4. How many years ago did you hear about autism spectrum disorder for the first time? (n = 433)	Last year	21	4.8
	2-4 years ago	135	31.1
	5-10 years ago	151	34.8
	10 + years ago	126	29.0
5. How would you evaluate your past interactions with people with autism spectrum disorder? (n = 434)	Positive	167	38.5
	Neutral	88	20.3
	Negative	6	1.4
	I don’t know	35	8.1
	I have not had any interaction with a person with autism	138	31.8

The type of personal experience (Item 2) was categorized as direct contact (e.g., being autistic, having a close friend or family member who is autistic, knowing an autistic individual, or working with autistic individuals) and indirect contact (e.g., exposure through media, books, word of mouth, or school/work). Scores for each type of experience were calculated by assigning values between 0 and 3 based on the number of selected items. The amount of experience was further assessed through the number of individuals encountered (Item 3) and the duration of familiarity with autism (Item 4). The number of individuals encountered included both direct interactions and indirect exposure (e.g., media representations) and was scored ordinally, ranging from 0 (no reported contact) to 5 (contact with 10 or more individuals). Similarly, duration of familiarity was scored from 0 (within the last year) to 3 (10 or more years).

To create a personal experience total score for the analyses, z-scores were obtained by standardizing the scores of direct experience, indirect experience, the number of different autistic people encountered, and the timing of first exposure to information about autism. These z-scores were then averaged to create a composite variable reflecting the amount and duration of personal experience [11]. Higher scores indicated greater personal experience. The Cronbach’s alpha coefficient for this section, consisting of four items, was .539. This relatively low reliability may reflect the small number of items and the heterogeneous nature of personal experience [54,55]. In such cases, mean inter-item correlation is considered a more appropriate indicator, with recommended values ranging between .2 and .4 [56]. In this study, the mean inter-item correlation was .23, indicating an acceptable level of internal consistency. In addition, this level of reliability has been considered acceptable in previous research [57–60]. Therefore, findings related to this variable should be interpreted with caution.

Finally, the quality of past interactions with autistic individuals (Item 5) was scored from 0 to 2 based on “negative,” “neutral,” and “positive” responses, excluding “I don’t know” and “no interaction” response options. As this variable was not significantly associated with the other components of personal experience (see Table 4), it was not included in the total score and was treated as a separate variable (interaction quality).

#### Knowledge.

The knowledge section of the questionnaire consisted of eight items, including three check-all-that-apply items and five multiple-choice questions. As shown in Table 3, one item from the original questionnaire (“How many children are currently thought to be on the autism spectrum in America?”) was not included, as comparable prevalence data are not yet clearly established in the Turkish context. Instead, participants were asked, “Which of the following statements about ASD is correct?”, adapted from Jones et al. [8]. In addition, an “I am not sure” response option was added to Items 1, 4, 6, 7, and 8 to reduce the likelihood of random guessing.

**Table 3:** Descriptive statistical results related to the knowledge level.

Item	Answer Options	f	%
1.When is autism spectrum disorder thought to start developing? (n = 507)	In the womb before birth^*^	443	87.4
	After vaccinations	18	3.6
	After head injury	8	1.6
	After another person being caught	4	0.8
	I am not sure	34	6.7
2. What are behaviors related to autism spectrum disorder? (Please mark all options that apply to you)	Clapping hands in front of face^*^	275	54.2
	Rocking back and forth^*^	359	70.8
	Talking to imaginary friends	70	13.8
	Repetitive words and phrases^*^	300	59.2
	Creating plans containing violence against peers	56	11
	Getting distressed in the presence of loud sounds^*^	398	78.5
3. In which areas do individuals with autism spectrum disorder experience difficulties? (Please mark all options that apply to you)	Making eye contact^*^	395	77.9
	*Physical growth*	53	10.5
	Establishing close relationships with peers^*^	388	76.5
	Verbal communication^*^	386	76.1
	Expressing yourself using art	73	14.4
4. Individuals diagnosed with autism spectrum disorder have intellectual disabilities, i.e., they have below-average intelligence levels	Correct	91	17.9
	Incorrect^*^	376	74.2
	I am not sure	40	7.9
5. Which of the following statements about autism spectrum disorder is correct? (Please mark all options that apply to you)	The number of individuals diagnosed with autism spectrum disorder in the world is increasing. ^*^	329	64.9
	Autism spectrum disorder impacts only males.	5	1.0
	*Autism spectrum disorder is a mental health problem.*	72	14.2
	Autism spectrum disorder impacts everyone differently. ^*^	278	54.8
6. Which of the following statements about autism spectrum disorder is the most correct?	Autism spectrum disorder cannot be treated, and the rate of an individual’s development can never be improved.	9	1.8
	Autism spectrum disorder cannot be treated, but individuals can increase their rates of development through meticulous education.^*^	359	70.8
	Autism spectrum disorder can be cured with a combination of medications and therapy/treatment.	114	22.5
	Autism spectrum disorder can be treated with a surgical operation on the brain.	6	1.2
	I am not sure	19	3.7
7. All individuals with autism spectrum disorder display aggressive behaviors such as hitting or biting.	Correct	117	23.1
	Incorrect^*^	360	71.0
	I am not sure	30	5.9
8. “Savant” skills are a symptom of autism, in other words, individuals with autism are highly skillful in a particular area and lack skills in other areas.	Correct	308	60.7
	Incorrect^*^	140	27.6
	I am not sure	59	11.6

*Correct answers. *Items in italics* were removed since they did not have distinctiveness while obtaining the total score.

To obtain a total score, each response option within multiple-response items was treated as a separate item, resulting in a total of 20 items. In the item analysis, two items presented in italics in Table 3 were excluded due to low item discrimination and negative item–total correlations. Following their removal, the final scale consisted of 18 items. The Cronbach’s alpha coefficient for the initial 20–item scale was .680, which increased to .707 after these items were excluded. Participants’ responses were coded dichotomously, with correct answers scored as 1 and incorrect answers scored as 0. Thus, the total score ranged from 0 to 14, with higher scores indicating greater knowledge about autistic characteristics and autism-related information.

#### Interaction Comfort.

The interaction comfort section of the questionnaire included three short video clips of autistic adults, followed by ten Likert-type items adapted from Jacoby [53]. The video stimuli were sourced from publicly available platforms (YouTube) and were originally selected during the questionnaire development process [53] based on several criteria. First, each clip depicted an autistic adult displaying observable characteristics commonly associated with autism, allowing participants to identify relevant behaviors. Second, the three individuals were deliberately selected to represent different presentations, ensuring exposure to a range of profiles rather than a single stereotypical representation. Third, the clips were brief and concise, making them suitable for use in a survey context. The durations of the clips were approximately 29, 17, and 22 seconds. Finally, the content of each video was aligned with the corresponding interaction scenarios, such that the questions reflected the perceived level of support needs and facilitated more realistic and contextually appropriate judgments. However, it should be noted that the original selection process did not follow a formal, systematic selection protocol. Future research may benefit from developing and validating video stimuli through more structured selection procedures.

For the purposes of this study, the individuals in the videos were categorized as having mild, moderate, and high support needs based on observable characteristics in the domains of social communication, restricted interests, and repetitive behaviors. The individual with mild support needs demonstrated verbal communication but showed difficulties in initiating and maintaining social interactions. The individual with moderate support needs was non-verbal but used assistive communication devices, with more limited social interaction. The individual with high support needs used limited verbal communication and displayed more noticeable behavioral responses (e.g., vocalizations or distress) in response to changes in routine or unfamiliar environments.

Because the original videos were in English, all spoken content was translated into Turkish through subtitles. After viewing each clip, participants responded to three or four items assessing how comfortable they would feel interacting with the individuals in different contexts. These contexts ranged from social to professional settings and varied in levels of familiarity and intimacy, consistent with the structure proposed by Jacoby [53]. The items were tailored to reflect the perceived level of support needs of the individuals in the videos and common adult life contexts. These contexts included both social and professional situations (e.g., recreational activities and workplace interactions), consistent with the structure proposed by Jacoby [53], and were intended to reflect realistic and contextually relevant interactions in everyday life. Responses were rated on a scale from 0 (very uncomfortable) to 10 (very comfortable), with 5 indicating a neutral level of comfort. The internal consistency of the ten items was high (Cronbach’s α = .921). Scores were averaged across items to obtain a composite interaction comfort score ranging from 0 to 10, with higher scores indicating greater comfort.

### Adaptation of the data collection tool and data collection process

The adaptation of the questionnaire followed a multi-step procedure. First, the original items were translated into Turkish and then back-translated into English by two independent bilingual translators to ensure linguistic equivalence. Discrepancies between the original and back-translated versions were reviewed and resolved by the researcher, ensuring semantic and conceptual equivalence between versions. Following this process, the questionnaire was evaluated by a panel of three experts, including two autism experts with experience in research and practice and one expert in measurement and evaluation, to assess the content validity of the items in terms of relevance, clarity, representativeness, and cultural appropriateness. Based on expert feedback, items considered less culturally appropriate (e.g., race/ethnicity) or for which reliable contextual data were not available in the Turkish context (e.g., prevalence estimates) were removed, and new items were added where necessary. In addition, response options were revised, and minor linguistic adjustments were made, as outlined in the data collection tool section above. Subsequently, the revised questionnaire was pilot-tested with five individuals representing the target population (aged 18 and above with varying educational levels) to examine item clarity, comprehensibility, and overall usability, thereby contributing evidence for face validity. Feedback from the pilot study informed minor revisions to the questionnaire, and following these refinements, the final version was established.

Data collection was carried out between May 1, 2021, and January 1, 2022. Participants from the seven geographical regions of Turkey were initially contacted via telephone, and the survey link was distributed through WhatsApp and e-mail. These participants were asked to forward the survey to other eligible individuals, and the survey was also disseminated through social media platforms such as Instagram. To minimize potential biases associated with online recruitment, participants were presented with an informed consent form prior to participation. The form stated that (a) the study had received ethical approval, (b) participation was voluntary and could be withdrawn at any time, (c) responses would be collected anonymously and used solely for research purposes, and (d) data would be collected through a secure online platform (SurveyMonkey). Participants provided consent before proceeding with the survey. To ensure eligibility, an option indicating “under 18 years of age” was included in the demographic section, and the survey was automatically terminated for those selecting this option.

### Data analysis

Data were analyzed using SPSS 26 and the PROCESS v4.3 macro [61]. Descriptive statistics were calculated to summarize participants’ responses. Inferential analyses were performed to examine differences in personal experience, knowledge, and interaction comfort across demographic variables. Independent-samples t-tests were used to examine differences by gender, type of residence, and relationship with special education. Paired-samples t-tests were conducted to compare within-participant differences, including direct versus indirect personal experience and interaction comfort across different contexts (e.g., social vs. professional settings). One-way ANOVA was conducted to examine differences by age, geographical region, and education level. The “no education level completed” category was excluded due to an insufficient number of participants (n = 1). Post hoc comparisons following significant ANOVA results were performed using Bonferroni correction. Pearson’s product–moment correlations (for continuous variables and z-scores) and Spearman’s rank-order correlations (for ordinal variables) were used to examine relationships among variables before conducting regression analyses. To control for Type I error associated with multiple comparisons, Bonferroni-adjusted p values < .05 were considered statistically significant.

Hierarchical regression analyses were conducted to examine predictive relationships among variables. Prior to these analyses, key assumptions were tested. The results indicated no evidence of multicollinearity (condition index < 30, tolerance > .10, variance inflation factor < 10, and intercorrelations below .80), no autocorrelation (Durbin-Watson values between 1 and 3), and linear relationships between variables. Based on the regression results and relevant literature, mediation analyses were conducted using the PROCESS macro. Compared to traditional regression approaches, PROCESS provides more robust estimates of indirect effects by accounting for shared variance between variables [62]. A simple mediation model (Model 4) was used to test whether knowledge (M) mediated the relationship between personal experience (X) and interaction comfort (Y), both with and without including interaction quality as a covariate (C). In addition, a moderated mediation model (Model 14) was tested to examine whether interaction quality (W) moderated the relationship between knowledge and interaction comfort.

## Results

### Findings regarding personal experience

According to Table 2, most participants reported having heard of ASD, primarily through indirect forms of contact. Indirect experiences (*M* = 1.98, *SD* = .71) were significantly more common than direct experiences (*M* = 1.20, *SD* = 1.11) (*t*_*(160)*_ = 13.93, *p* < .001).

Personal experience did not differ by gender (*t*_*(505)*_ = .760, *p* = .448), but was higher among urban participants (*M* = .03, *SD* = 1.00) than rural participants (*M* = −.30, *SD* = .85) and among individuals with a relationship to special education (*M* = .60, *SD* = 1.03) than those without such a relationship (*M* = −.23, *SD* = .87) (*t*_*(505)*_ = 2.234, *p* = .026; *t*_*(505)*_ = .920, *p* < .001).

Personal experience also differed by age, geographical region, and educational level (*F*_*(4,502*_*)* = 3.196, *p* = .013; *F*_*(6,500)*_ = 2.851, *p* = .010; *F*_*(6,499)*_ = 4.728, *p* < .001), with higher scores among participants aged 28–38 (*M* = .133, *SD* = 1.057) compared to those aged 18−27 (*M* = −.233, *SD* = .958) (*p* = .009), among those from the Mediterranean region (*M* = .383, *SD* = 1.03) compared to Eastern Anatolia (*M* = −.146, *SD* = .938) (*p* = .044), and among university participants (*M* = .101, *SD* = 1.053) and participants with master’s degrees (*M* = .178, *SD* = .969) compared to high school graduates (*M* = −.391, *SD* = .896) (*p* = .002; *p* = .003).

### Findings regarding knowledge

Considering skewness (−.974, *SE* = .108) and kurtosis (.906, *SE* = .217), knowledge scores were normally distributed. Measures of central tendency (*M* = 9.43, *Median* = 10, *Mode* = 12, *SD* = 2.85, *Range* = 0–14) indicated a slightly left-skewed distribution and relatively high knowledge levels. A small proportion of participants answered all items correctly (1.8%, *n* = 9), while 1.4% (*n* = 7) answered all items incorrectly (see Table 3).

Knowledge differed significantly by gender, residence, and relationship to special education. Female participants (*M* = 9.77, *SD* = 2.73) scored higher than males (*M* = 8.79, *SD* = 2.95) (*t*_*(505)*_ = 3.69, p < .001). Urban participants (*M* = 9.60, *SD* = 2.73) had higher knowledge levels than rural participants (*M* = 7.82, *SD* = 3.45) (*t*_*(505)*_ = 4.130, *p* < .001). Similarly, individuals wi*t*h a relationship to special education (*M* = 10.44, *SD* = 2.50) scored higher than those without such a relationship (*M* = 9.04, *SD* = 2.88) (*t(505)* = 5.112, *p* < .001).

Knowledge did not differ by age (*F*_*(4,502)*_ = 1.81, *p* = .125), but differed by geographical region and educational level (*F*_*(6,500)*_ = 2.643, *p* = .016; *F*_*(6,499)*_ = 3.997, *p* = .001). Post hoc analyses showed that participants from the Marmara region (*M* = 10.150, *SD* = 2.268) scored higher than those from Eastern Anatolia (*M* = 9.021, *SD* = 2.940) (*p* = .044). In terms of education, primary school graduates (*M* = 5.250, *SD* = 3.011) had significantly lower knowledge levels than high school graduates (*M* = 9.023, *SD* = 3.127), vocational high school graduates (*M* = 9.095, *SD* = 2.624), university graduates (*M* = 9.552, *SD* = 2.842), master’s degree holders (*M* = 9.903, *SD* = 2.075), and participants with doctoral degrees (*M* = 9.719, *SD* = 2.907) (*p* = .006; *p* = .020; *p* < .001; *p* < .001; *p* = .001).

### Findings regarding interaction comfort

The mean interaction comfort score was 6.61 (*SD* = 1.98, *range* = 0−10). The distribution was approximately normal (skewness = −.312, *SE* = .108; *kurtosis* = −.244, *SE* = .217). In total, 36.5% of participants reported moderate levels of comfort, while 3.4% reported very low levels of comfort and 15.2% reported very high levels of comfort. Interaction comfort differed according to levels of support needs, with lower scores for individuals with high support needs (*M* = 5.92, *SD* = 2.23) compared to those with moderate (*M* = 6.61, *SD* = 2.25) and low support needs (*M* = 7.51, *SD* = 2.29).

Participants reported higher interaction comfort in professional contexts, such as being a cashier, waiter, colleague, or doctor (*M* = 6.74, *SD* = 2.08) than in social contexts, such as going out to dinner, having mutual friends, and being neighbors with autistic individuals (*M* = 6.52, *SD* = 2.02) (*M* = .224, *SD* = .99; *t*_*(506)*_ = 5.06, *p* < .001). The highest-rated item was interaction with a neighbor with low support needs (*M* = 7.56, *SD* = 2.47), whereas the lowest-rated item was having a one-on-one meal with an individual with high support needs (*M* = 5.40, *SD* = 2.71).

Interaction comfort did not differ by gender (*t*_*(505)*_ = −.25, *p* = .797), residence (*t*_*(505)*_ = 0.85, *p* = .932), or relationship with special education (*t*_*(505)*_ = 1.751, *p* = .081). However, a significant difference was observed across age groups (*F*_*(4,502)*_ = 3.695, *p* = .006), but not across geographical region or education level (*F*_*(6,500)*_ = 1.931, *p* = .074; *F*_*(6,499)*_ = 1.577, *p* = .152). Post hoc analyses indicated higher scores for participants aged 28–38 (*M* = 6.938, *SD* = 1.991) compared to those aged 39–49 (*M* = 6.150, *SD* = 1.936) (*p* = .005).

### Findings regarding predictive and mediating relationships between personal experience, knowledge level, and interaction comfort

Table 4 shows that interaction comfort with individuals with different levels of support needs was positively correlated with personal experience, interaction quality, and knowledge, although the magnitude of these relationships was small (*r* < .30). Despite their small magnitude, all correlations remained statistically significant after Bonferroni correction. Given these statistically significant associations, further analyses were conducted to examine the predictive roles of these variables. Hierarchical regression analyses were conducted to examine whether these variables predicted interaction comfort, and the results are presented in Table 5.

**Table 4:** Correlations among personal experience, knowledge, and interaction comfort.

	Direct experience	Indirect experience	Number of people with a known diagnosis	Years when ASD is known	Quality of interaction	Total experience (*z*)	Knowledge	Interaction comfort	High support needs	Moderate support needs	Low support needs
Direct experience (*z*)	1	**.158** ^**^	**.148** ^**^	.034	−.031	**.397** ^**^	.111^*^	.095^*s^	.077	.076	.087^*^
Indirect experience (*z*)		1	**.163** ^**^	**.160** ^**^	.021	**.608** ^**^	**.213** ^**^	.080^s^	.084	.072	.051
Number of people with a known diagnosis (*z*)			1	**.233** ^**^	.058	**.691** ^**^	**.313** ^**^	**.169** ^**s^	**.149** ^**s^	.127^**s^	**.157** ^**s^
Years when ASD is known (z)				1	−.015	**.654** ^**^	.131^**^	.090^*s^	.134^**s^	.020	.053
Quality of interaction					1	.017	.084	**.233** ^**s^	**.277** ^**s^	.179^**s^	.112
Total personal experience (z)						1	**.330** ^**^	**.151** ^**s^	**.161** ^**s^	.091^**s^	.124^**s^
Knowledge							1	**.235** ^**s^	**.173** ^**s^	**.224** ^**s^	**.208** ^**s^
Interaction comfort								1			

* p < .05, ** p < .01; Pearson’s correlations are reported for continuous variables, and Spearman’s rank-order correlations are reported for ordinal variables. z = standardized (z) score.

Note: Bold values indicate significance after Bonferroni correction.

**Table 5:** Results of the hierarchical regression analysis for interaction comfort.

	Model 1				Model 2				Model 3			
	B	SE B	β	*t*	B	SE B	β	*t*	B	SE B	β	*t*
PE	3.01	1.15	.15	2.62^**^	2.93	1.11	.15	2.62^**^	1.64	1.18	.08	1.38
QI					8.48	2.04	.23	4.159^***^	7.98	2.01	.22	3.95^***^
Knowledge									1.33	.45	.18	2.95^**^
R^2^	.024 .024 6.864^**^				.081 .057 17.299^***^				.109 .028 8.703^**^			
R^2^_change_												
*F*_change_												

*p < .05, **p < .01, ***p < .001; PE: Personal Experience; QI: Quality of Interaction

The results of the hierarchical regression analysis showed that personal experience was no longer a significant predictor of interaction comfort after the inclusion of knowledge, suggesting that knowledge may mediate the relationship between personal experience and interaction comfort. Accordingly, knowledge was tested as a mediator, with interaction quality included as a covariate, and the results of Model 4 are presented in Table 6.

**Table 6:** The mediator role of knowledge in the relationship between personal experience and interaction comfort.

	Total Effect (p)	Direct Effect (p)	Indirect Effect	Confidence Intervals	t	Result
*Model 1* Personal Experience→Knowledge→Interaction Comfort	2.97 (000)	1.76 (.053)	1.20	.549/1.93	3.34	Significant indirect effect
*Model 2* Personal Experience→Knowledge→Interaction Comfort	2.93 (.009)	1.64 (.167)	1.29	.390/2.35	3.31	Significant indirect effect

Note: In Model 1, the quality of interaction was not included as a covariate variable. In Model 2, the quality of interaction was included in the model as a covariate variable related to interaction comfort.

The mediation analysis (Model 4) indicated that the indirect effect of personal experience on interaction comfort through knowledge was significant, both without (Model 1) and with the inclusion of interaction quality as a covariate (Model 2; see Table 6). In both models, the direct effect of personal experience was not significant after knowledge was included in the model. The magnitude of the indirect effect appeared to increase when interaction quality was included as a covariate. Given that interaction comfort varied across different levels of support needs, additional analyses were conducted to examine whether the predictors of interaction comfort differed across these levels. The results of these analyses are presented in Table 7.

**Table 7:** Results of the hierarchical regression analysis of interaction comfort across different levels of support needs.

		Model 1				Model 2				Model 3			
		B	SE B	β	*t*	B	SE B	β	*t*	B	SE B	β	*t*
High support needs	PE	.279	.135	.123	2.06^*^	.188	.145	.083	1.29	.198	.139	.087	1.42
	Knowledge					.094	.055	.109	1.71	.073	.053	.084	1.37
	QI									1.127	.237	.272	4.75^***^
	R^2^	.015 .015 4.282^*^				.025 .010 2.937				.099 .073 22.630^***^			
	R^2^_change_												
	*F*_change_												
		**Model 1**				**Model 2**				**Model 3**			
Moderate support needs	PE	.268	.129	.123	2.08^*^	.262	.127	.120	2.06^*^	.107	.135	.049	.79
	QI					.737	.231	.186	3.18^**^	.677	.229	.171	2.96^**^
	Knowledge									.159	.051	.193	3.10^**^
	R^2^	.015 .015 4.326^*^				.050 .035 10.151^**^				.082 .032 9.659^**^			
	R^2^_change_												
	*F*_change_												
		**Model 1**				**Model 2**							
Low support needs	PE	.286	.101	.125	2.83^**^	.147	.106	.064	1.39				
	Knowledge					.148	.037	.184	4.00^***^				
	R^2^	.016 .016 8.014^**^				.046 .030 16.003^***^							
	R^2^_change_												
	*F*_change_												

* p < .05, ** p < .01, *** p < .001; PE: Personal Experience; QI: Quality of Interaction

In the final models, different patterns of association emerged across levels of support needs. For individuals with low support needs, knowledge was significantly associated with interaction comfort. For those with moderate support needs, both knowledge and interaction quality were associated with interaction comfort, although the association with interaction quality did not remain significant after Bonferroni correction. For individuals with high support needs, interaction quality was significantly associated with interaction comfort, as shown in Table 7.

These findings suggest that knowledge may mediate the relationship between personal experience and interaction comfort, and that interaction quality may play a moderating role in the relationship between knowledge and interaction comfort across different levels of support needs. Mediation analysis (Model 4) indicated that the indirect effect of knowledge was significant (direct effect: *b* = .258, *t* = 2.49, *p* = .013; indirect effect: *b* = .088, *t* = 2.33, *95% CI* [.054, .462]). Moderated mediation analysis (Model 14) showed that the indirect effect was significant at higher levels of interaction quality, as presented in Table 8. In addition, the index of moderated mediation was significant (see Table 8), suggesting the presence of a moderated mediation effect.

**Table 8:** Moderated mediation analysis of the indirect effect of personal experience on interaction comfort.

Direct Relationship	Unstandardized Coefficient		t values	
Personal Experience→ Knowledge	0.9747		6.64	
Personal Experience→ Interaction Comfort	0.1546		1.1034	
Knowledge→ Interaction Comfort	0.0827		1.5640	
Knowledge*Quality of Interaction→ Interaction Comfort	0.1670		2.0539	
Indirect Relationship	Direct Effect	Indirect effect (SE)	95% CI	T values
Personal Experience →Knowledge→Interaction Comfort	0.1546	0.0806 (0.0517)	−.0209/.1815	1.5589
Probing Moderated Indirect Relationship	Effect	SE	95% CI	t-statistic
Low interaction quality	−0.0080	0.0666	−0.1481/0.1169	0.120
High interaction quality	0.1446	0.0629	0.0232/0.2704	2.298
Index of Moderated Mediation	0.1627	0.0833	0.0084/0.3410	1.9531

**Fig 1:**
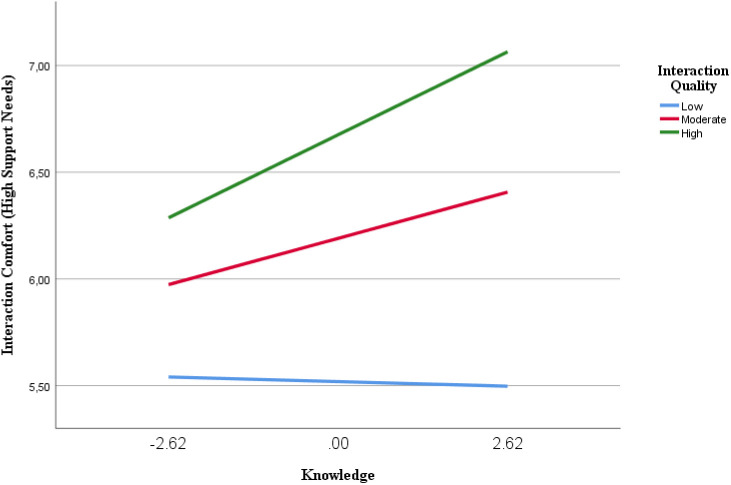
The moderating effect of interaction quality on the relationship between knowledge and interaction comfort.

A simple slope analysis suggested that higher interaction quality was associated with a stronger positive relationship between knowledge and interaction comfort (see Fig 1).

## Discussion

### Levels of personal experience, knowledge, and interaction comfort

The present study examined Turkish adults’ personal experiences with autistic individuals, their knowledge of autism, and their interaction comfort, as well as whether these variables varied across demographic characteristics and how they were related to one another. Consistent with findings from other cultural contexts [2,3,6,8,11], most participants reported having heard of ASD. Experiences were predominantly indirect, shaped largely by social media and educational contexts [2,11,63], although most participants had encountered at least one autistic individual within the past 5–10 years, and direct experiences were generally positive. Participants demonstrated relatively high levels of knowledge, consistent with previous research [3,6,11,51]. They showed an accurate understanding of commonly recognized autistic characteristics [63], including recognizing that intellectual disability is not a defining characteristic, although some misconceptions persisted, particularly regarding savant skills and medical treatment. Interaction comfort was close to the midpoint and varied by both support needs and context, with lower levels reported for individuals with higher support needs and in social settings, and higher levels in professional contexts. However, these findings should be interpreted cautiously, as some participants did not respond to the interaction comfort items.

These findings may reflect broader contextual developments in Turkey. Increasing reported prevalence rates, the National Autism Action Plan introduced in 2016, and the growing visibility of autism through governmental and non-governmental initiatives, awareness campaigns, and media content may have contributed to greater public awareness and increased opportunities for both direct and indirect encounters. The inclusion of special education-related content in university curricula may have further supported exposure, particularly among individuals with relatively higher levels of education. However, these patterns should be interpreted in light of the characteristics of the study sample. The use of online data collection and snowball sampling, together with the relatively high educational level of participants, may have increased the likelihood of participation among individuals already familiar with or interested in ASD. Therefore, the relatively high levels of awareness and indirect familiarity observed in this study may not fully represent the broader Turkish population.

Despite these developments, a considerable proportion of participants reported no direct interaction with autistic individuals, indicating a persistent gap between awareness and direct social experience. While indirect exposure—especially through media and educational contexts—may increase familiarity, it does not necessarily translate into meaningful interpersonal interactions. This suggests that current efforts in Turkey may have been more effective in raising awareness than in fostering more inclusive social interactions. However, this pattern should also be interpreted with caution, as the internal consistency of the personal experience subscale was relatively low, which may have influenced the interpretation of these findings.

Consistent with this pattern, participants demonstrated relatively high levels of knowledge alongside the persistence of specific misconceptions. The widespread belief regarding savant skills may be linked to media representations of autism, as films and television series (e.g., Rain Man, A Miracle Doctor) often emphasize exceptional abilities and more stereotypical portrayals [64]. As noted by Dillenburger et al. [6], such representations may simultaneously promote awareness and contribute to misconceptions, leading to oversimplified or partially distorted understandings. In contrast, the consistent emphasis on social communication differences and restricted or repetitive behaviors across contexts [64] may have supported more accurate identification of commonly recognized autistic characteristics.

A similar pattern emerged in interaction comfort. Reported comfort levels may reflect contextual and methodological influences rather than broader or stable attitudes, particularly given the use of video-based scenarios and the absence of direct interaction [11,65]. Non-response patterns further suggest uncertainty in evaluating interaction situations. Moreover, lower comfort levels in interactions involving individuals with greater support needs indicate that knowledge alone may not translate into interaction comfort, especially in more socially demanding contexts. Interaction comfort also varied by setting, with higher levels reported in professional contexts than in social settings. This finding contrasts with previous research conducted in the United States [11] and may reflect cultural and contextual differences. In the Turkish context, which is characterized by more collectivist social norms, social interactions often involve implicit expectations regarding socially appropriate behavior and a heightened concern about making mistakes or violating social norms. Such uncertainty may reduce individuals’ comfort when interacting with autistic individuals in informal social settings. In contrast, professional environments may provide clearer roles, expectations, and institutional support, which can reduce ambiguity and facilitate more structured and predictable interactions, thereby increasing interaction comfort. This pattern is also consistent with contact theory [29], which suggests that more positive intergroup attitudes are more likely to develop under conditions such as equal status, shared goals, cooperation, and institutional support [34]. Accordingly, not only the presence of contact but also its quality and contextual features appear to be particularly important.

### Demographic differences in personal experience, knowledge, and interaction comfort

The findings indicate that variation across demographic characteristics was more pronounced in personal experience, whereas it was more limited for knowledge and interaction comfort. This more pronounced variation in personal experience suggests that opportunities for encountering and engaging with autistic individuals are not equally distributed in the Turkish context. Such variation may be related to differences in contact opportunities shaped by individuals’ living environments, educational backgrounds, and access to services. In Turkey, the concentration of special education services and autism-related services and support systems in urban areas may increase opportunities for encountering autistic individuals, particularly for those living in large cities and in the Marmara region. In contrast, in regions where services and awareness are relatively limited, such opportunities for contact may be more restricted, which may, in turn, limit opportunities for meaningful intergroup contact as emphasized in contact theory.

The finding that women demonstrated higher levels of knowledge is consistent with previous research across different cultural contexts [6,42,43,66]. This may be explained by women’s greater interest in autism- and disability-related topics [8,42,51] and their more frequent engagement in caregiving roles shaped by gender norms, which may contribute to greater opportunities for contact with autistic individuals [43,63]. However, this finding should be interpreted with caution, as the sample was predominantly composed of women, and higher levels of education were associated with increased knowledge, consistent with prior research [2,3,6,8,43,51,66], which may reflect greater exposure to autism-related information in academic contexts [67]. Similarly, higher levels of knowledge among individuals living in urban areas and in the Marmara region may be linked to easier access to information sources and services. In contrast, the lack of age-related differences in knowledge is consistent with mixed findings in the literature [3,6,8,24,43,51,66], and may reflect the increasing availability of autism-related information across age groups through media visibility and digital platforms in recent years.

The limited variation in interaction comfort across demographic characteristics suggests that this variable cannot be explained solely by individual-level factors. This finding contrasts with studies reporting demographic differences in attitudes [8,43,48,50,51,63,68]. However, it may be related to the limited availability of direct and meaningful interaction opportunities with autistic individuals in the Turkish context. The fact that different demographic groups reported similar levels of interaction comfort may indicate that they share similarly limited experiences in terms of encountering and interacting with autistic individuals. In this sense, interaction comfort appears to be more sensitive to individuals’ real-life experiences and the quality of interactions than to demographic characteristics. In other words, having knowledge about autism alone may not be sufficient for individuals to feel comfortable in social interactions. The finding that relatively younger participants reported higher interaction comfort is partially consistent with previous research [69] and may be associated with younger generations’ greater openness to inclusion, diversity, and individual differences.

### Predictors of interaction comfort and indirect relationships

Consistent with previous research [2], the findings of this study indicate that the effect of personal experience on interaction comfort operates indirectly through knowledge rather than occurring directly. This suggests that mere contact with autistic individuals may not be sufficient to enhance interaction comfort; rather, how these experiences are interpreted may plays an important role. This finding is consistent with contact theory [29], which emphasizes that the impact of contact depends not only on its occurrence but also on how it is cognitively processed. In this sense, personal experience provides opportunities for contact, while knowledge may help individuals to interpret these experiences, which may contribute to greater interaction comfort.

The findings further indicate that, in line with the model tested in this study, interaction comfort may not reflect a purely unidirectional process but rather emerge from potentially reinforcing relationships among personal experience, knowledge, and interaction quality. In this regard, individuals’ knowledge may shape how they interpret interaction experiences, while the quality of these interactions, in turn, may influence how future experiences are perceived and understood.

Particularly in interactions involving individuals with higher support needs, experience alone may be insufficient; rather, these experiences may need to be supported by adequate knowledge and accompanied by positive interaction conditions. Moreover, the finding that the effect of personal experience on interaction comfort was stronger under more positive interaction conditions suggests that the variables in the model may operate in potentially reinforcing ways rather than as fully independent processes.

Taken together, these findings indicate that although awareness and knowledge about ASD have increased in Turkey, this progress may not fully translate consistently into individuals’ everyday interactions with autistic individuals. Examining the relationship among personal experience, knowledge, and interaction comfort reveals that opportunities for contact and access to information vary across demographic and contextual factors, yet these differences do not directly translate into interaction comfort. This suggests that interaction comfort cannot be explained solely by levels of knowledge or the amount of experience, but rather may reflect a more complex and context-dependent process. Current efforts in Turkey appear to focus primarily on raising awareness and increasing knowledge, while structured opportunities for direct, meaningful, and high-quality interaction with autistic individuals may remain limited. Therefore, fostering more inclusive social environments may require not only increasing awareness but also promoting the integration of knowledge with real-life experiences and expanding structured opportunities that support positive and meaningful interaction.

### Limitations and future research directions

This study has several limitations that should be considered when interpreting the findings. First, the use of online data collection and snowball sampling may have resulted in a sample that was not fully representative of the broader population, particularly given the relatively high educational level of participants, the predominance of female respondents, and the concentration of participants in specific regions (especially Eastern Anatolia). Second, the internal consistency of the personal experience subscale was relatively low, which may have influenced the interpretation of the related findings. In addition, as the data were based on self-reports and closed-ended survey items, participants’ responses may have been influenced by response bias and may not fully reflect the complexity of their experiences. Third, the use of video-based scenarios instead of direct interaction may have influenced participants’ responses regarding interaction comfort, potentially limiting ecological validity [11,65]. Furthermore, the cross-sectional design of the study does not permit causal interpretations of the relationships among personal experience, knowledge, and interaction comfort.

Future research should adopt longitudinal and experimental designs to better examine the directionality and potentially reciprocal nature of these relationships. In addition, studies with more diverse and representative samples, as well as those conducted in real-life interaction contexts, would provide a more comprehensive understanding of how interaction comfort develops. In particular, future studies may examine experimentally the effects of structured interaction opportunities across different contexts, such as schools, workplaces, and community settings (e.g., peer-mediated interaction programs and community-based engagement initiatives). Moreover, interventions designed to enhance the quality of direct interaction experiences with autistic individuals (e.g., guided interaction sessions or training-based programs) may be evaluated regarding their potential impact on interaction quality and social participation.

## Conclusion

This study contributes to the literature by examining Turkish adults’ personal experiences, knowledge, and interaction comfort regarding autistic individuals and by identifying the relationships among these variables. The findings indicate that although awareness and knowledge about ASD are relatively high, these do not necessarily translate into confident and meaningful social interactions. Instead, interaction comfort appears to reflect a more complex process shaped by the interplay of personal experience, knowledge, and interaction quality.

These findings provide insights into the current situation in Turkey. While increasing public awareness through policy initiatives and societal efforts may have contributed to improving general knowledge about autism, this progress may not appear to be accompanied by a corresponding increase in direct and meaningful interaction experiences. This suggests that knowledge alone may be insufficient to facilitate positive social engagement, particularly in contexts involving higher support needs or less structured interaction settings.

Accordingly, these results highlight the need to move beyond awareness-raising efforts toward approaches that promote both the quality and frequency of meaningful interaction experiences. Supporting structured and inclusive opportunities across educational, professional, and community settings may be particularly important for fostering more confident, inclusive, and reciprocal social engagement with autistic individuals. In this context, developing and disseminating structured programs aimed at increasing direct and high-quality interaction opportunities may play an important role in supporting more inclusive and sustainable social engagement.
